# Diagnostic test accuracy of D-dimer for acute aortic syndrome: systematic review and meta-analysis of 22 studies with 5000 subjects

**DOI:** 10.1038/srep26893

**Published:** 2016-05-27

**Authors:** Hiroki Watanabe, Nobuyuki Horita, Yuji Shibata, Shintaro Minegishi, Erika Ota, Takeshi Kaneko

**Affiliations:** 1 Department of Pulmonology, Yokohama City University Graduate School of Medicine, Yokohama, Japan; 2 Department of Cardiology, Yokohama City University Graduate School of Medicine, Yokohama, Japan; 3 Department of Health Policy, National Center for Child Health and Development, Tokyo, Japan

## Abstract

Diagnostic test accuracy of D-dimer for acute aortic dissection (AAD) has not been evaluated by meta-analysis with the bivariate model methodology. Four databases were electrically searched. We included both case-control and cohort studies that could provide sufficient data concerning both sensitivity and specificity of D-dimer for AAD. Non-English language articles and conference abstract were allowed. Intramural hematoma and penetrating aortic ulcer were regarded as AAD. Based on 22 eligible articles consisting of 1140 AAD subjects and 3860 non-AAD subjects, the diagnostic odds ratio was 28.5 (95% CI 17.6–46.3, I^2^ = 17.4%) and the area under curve was 0.946 (95% CI 0.903–0.994). Based on 833 AAD subjects and 1994 non-AAD subjects constituting 12 studies that used the cutoff value of 500 ng/ml, the sensitivity was 0.952 (95% CI 0.901–0.978), the specificity was 0.604 (95% CI 0.485–0.712), positive likelihood ratio was 2.4 (95% CI 1.8–3.3), and negative likelihood ratio was 0.079 (95% CI 0.036–0.172). Sensitivity analysis using data of three high-quality studies almost replicated these results. In conclusion, D-dimer has very good overall accuracy. D-dimer <500 ng/ml largely decreases the possibility of AAD. D-dimer >500 ng/ml moderately increases the possibility of AAD.

Acute aortic dissection (AAD) is one of the most dangerous emergency diseases^1^. Clinicians usually rank AAD as the top differential diagnosis for patients who present typical sudden onset severe chest or back pain. However, some AAD cases may present with very vague symptoms such as abdominal discomfort or syncope^2^. Although some imaging studies, namely enhanced computed tomography (CT), magnetic resonance angiography, transesophageal echocardiogram, and angiography, have high accuracy in diagnosing AAD, it is often difficult to perform these tests as an initial diagnostic test because of high-cost, radiation exposure from unnecessary tests, the limited resources available at a facility, and risks for anaphylaxis and acute kidney injury. Thus, we need a simple laboratory test to rule out AAD^3^,^4^.

D-dimer is a typical degradation product of cross-linked fibrin^5^, whose serum level is known to be a good diagnostic tool for deep venous thrombosis, pulmonary embolism, and AAD. For rule-out purposes, a cut off value should be set for high sensitivity. Based on research in pulmonary embolism, the cutoff value of 500 ng/ml is often chosen to rule out AAD^5^,^6^,^7^,^8^,^9^,^10^.

During the last 10 years, an increasing number of case-control and observational studies have reported the diagnostic accuracy of D-dimer for AAD^11^,^12^,^13^,^14^,^15^,^16^,^17^,^18^,^19^,^20^,^21^,^22^,^23^,^24^,^25^,^26^,^27^,^28^,^29^,^30^,^31^,^32^. In addition, a few systematic reviews and meta-analyses have calculated the pooled sensitivity and specificity using a univariate fixed- or random-model^5^,^6^,^7^,^8^,^9^,^10^,^27^. However, few original studies were included in these systematic reviews. In addition, the recent meta-analysis methodology for the diagnostic test accuracy strongly recommends the use of the hierarchical model instead of the separate pooling of sensitivity and specificity^33^,^34^,^35^,^36^. Thus, we conducted this systematic review and meta-analysis to assess the diagnostic accuracy of D-dimer for AAD based on the hierarchical model and a careful study search strategy^9^.

## Methods

### Study registration

The protocol was registered at the international prospective register of systematic reviews (PROSPERO) website on number CRD42015030095^37^. This study followed PRISMA statement and the Cochrane Handbook for Diagnostic Test Accuracy Reviews^34^,^38^.

Institutional review board ethical approval was not needed because of the reviewing nature of this study.

### Inclusion criteria and study search

We included case-control studies, prospective and retrospective cohort studies that could provide sufficient data concerning both sensitivity and specificity of D-dimer for AAD. Imaging modality as reference test should be clearly indicated in each study. Single- and two-gate studies are customarily termed cohort and case-control studies. Non-English language reports and conference abstracts were allowed.

### Study search

In the electronic search, we systematically searched Pubmed, EMBASE, the Cochrane Library advanced search, and Web of Science Core Collection. The search formulas are shown in the e-Appendix 1. References of previously published reviews and those of included original studies were checked through a hand search.

Two investigators independently screened the candidate articles by checking the title and abstract. After independent screening, articles that were still regarded as candidates by at least one investigator were then scrutinized independently through full-text reading. Final inclusion was decided after resolving discrepancies between the two investigators.

### Outcome

We considered blood D-dimer level as an index test measured by any method^3^,^4^. A cutoff value for D-dimer was set at 500 ng/ml, in accordance with previous meta-analyses^6^,^7^,^9^,^27^.

To diagnose AAD, the following exams were considered preferable reference tests: angiography, enhanced CT, CT angiography, magnetic resonance imaging, and trans-esophageal echocardiography^3^,^4^. Diagnosis by autopsy was also allowed. Any type of AAD according to the DeBakey classification and the Stanford classification were regarded as AAD. Aortic aneurism rupture, aortic aneurism pending rupture, and chronic aortic dissection were not considered as AAD^3^. Along with classic AAD, we regarded intramural hematoma, and penetrating aortic ulcer as AAD because it is clinically very difficult to distinguish them from classic AAD^3^.

Data that were extracted by the two investigators were crosschecked. First, we made two by two contingency tables from the number of subjects with true positives/false negatives/false positives/true negatives described in each original study. Then, we assessed diagnostic odds ratio (DOR), area under hierarchical summary receiver operating characteristic (HSROC) curve (AUC) to discover the overall diagnostic accuracy. The summary estimates of the sensitivity, the specificity, the positive likelihood ratio (PLR), the negative likelihood ratio (NLR), the positive predictive value (PPV) and the negative predictive value (NPV) were also assessed^33^.

### Quality assessment for bias and applicability

The two investigators independently evaluated each study by scoring seven domains of A Revised Tool for the Quality Assessment of Diagnostic Accuracy Studies (QUADAS-2) evaluation sheet^39^. Any discrepancies were resolved through discussion. Use of plain CT or trans-thoracic echocardiography led to high applicability concern for reference test. In this review, a “high-quality report” was defined as an original study that had neither high risk of bias nor high applicability concerns in any of the QUADAS-2 domains and that used a cut off value of 500 ng/ml. We used a high-quality report subgroup for sensitivity analyses.

### Data synthesis

We used both the HSROC model and bivariate model^33^,^34^,^35^,^36^. To determine the overall accuracy, we calculated the DOR using the DerSimonian-Laird random-effect model and the AUC using Holling’s proportional hazard models and using data from all studies with any D-dimer cutoff value^40^,^41^. A cutoff value of 500 ng/ml was used to estimate the summary estimates of the sensitivity, the specificity, PLR, NLR, PPV, and NPV. We obtained a paired forest plot, HSROC curve, and summary estimates of the sensitivity and the specificity using the bivariate model^34^. PLR and NLR were obtained using the summary estimate of the sensitivity and the specificity^34^. We estimated PPV and NPV across the pre-test probabilities in the range of 0–100%.

### Software

We drew a paired forest plot using Reviewing Manager ver. 5.3 (Cochrane Collaboration, Oxford, UK). The following commands of the “mada” package of the free software R were used: “madauni” for DOR, “phm” for AUC, and “reitsma” for the HSROC curve and the summary estimates for the sensitivity and the specificity^40^,^41^.

### Sensitivity analysis

We conducted two subgroup analyses: studies using the D-dimer cutoff value of 500 ng/ml and high-quality reports. The primary outcomes of this systematic review were from the main analysis and the results from the sensitivity analysis were used for judging the robustness of the main outcomes.

## Results

### Study search and study characteristics

**Figure 1:**
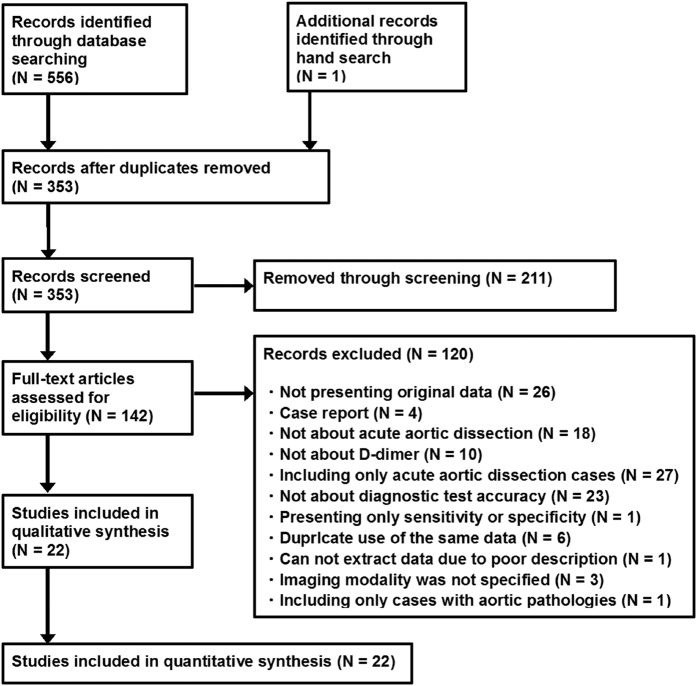
PRISMA flow chart for study search.

**Figure 2:**
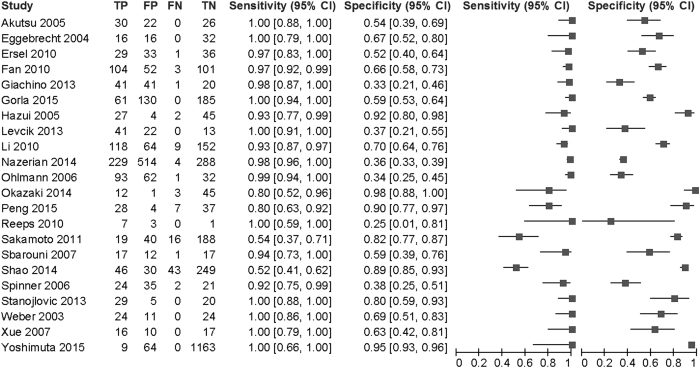
A paired forest plot by D-dimer for acute aortic dissection. TP: true positive. FP: false positive. FN: false negative. TN: true negative.

Of 557 articles that met the preliminary criteria, 204, 211, and 120 were excluded through removal of duplication, title/abstract screening, and full-article scrutinizing, respectively (Fig. 1). We finally found 22 eligible articles^11^,^12^,^13^,^14^,^15^,^16^,^17^,^18^,^19^,^20^,^21^,^22^,^23^,^24^,^25^,^26^,^27^,^28^,^29^,^30^,^31^,^32^. Most studies were from Asian and European countries (Table 1). The 22 articles included 14 cohort studies, eight case-control studies, two conference abstracts, and 20 full-length articles. One article was written in German and the others were written in English. The participants included in the studies ranged from 11 to 1236 with a median of 80. The total number of subjects was 5000, which consisted of 1140 AAD cases and 3860 controls. Across 22 studies, the sensitivity ranged from 0.52 to 1 with a median of 0.97 (IQR: 0.93–1.00) and the specificity ranged from 0.25 to 0.98 with a median of 0.64 (IQR: 0.37–0.97) (Fig. 2).

Nineteen studies had at least one domain of high risk of bias or high applicability concern, the other three had none. These three studies were classified as high-quality reports (Table 1, e-Fig. 1). Frequent causes of the high risk of bias and the high applicability concern were case-control study design, inappropriate subject recruiting, arbitrary selection of D-dimer cutoff value, and potential use of plain CT and trans-thoracic echocardiography as reference test.

### Overall diagnostic accuracy

**Figure 3:**
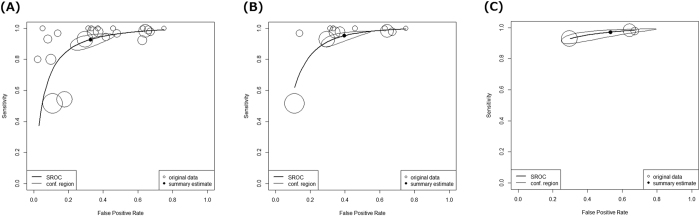
Hierarchical summary receiver operating characteristic curves by D-dimer for acute aortic dissection. (**A**) All studies regardless of the cutoff values (22 studies). (**B**) Studies with the cutoff value of 500 ng/ml (12 studies). (**C**) High-quality reports (three studies). Circle sizes suggest weights of diagnostic odds ratio in each study, not confidence regions.

Using data from all 22 studies consisting of 1140 AAD subjects and 3860 non-AAD subjects, DOR was 28.5 (95% confidence interval (95% CI) 17.6–46.3, I^2^ = 17.4%) and AUC was 0.946 (95% CI 0.903–0.994) (Table 2, Fig. 3A). According to a criterion of Jones *et al.*, AUC in the range of 0.93–0.96 was categorized as “very good” from the categories “excellent,” “very good,” “good,” and “reasonable”^42^.

According to the first sensitivity analysis using data from 12 studies that used the cutoff value of 500 ng/ml, DOR was 30.7 (95% CI 17.0–55.2, I^2^ = 7.7%) and AUC was 0.950 (95% CI 0.847–1.000) (Table 2, Fig. 3B). For the second sensitivity analysis, we evaluated three high-quality reports, which revealed DOR of 30.4 (95% CI 17.2–53.7, I^2^ = 0%) and AUC was 0.954 (95% CI 0.909–1.000) (Table 2, Fig. 3C). These values suggested that overall diagnostic accuracy did not change through sensitivity analyses.

### Sensitivity and specificity

Based on 833 AAD patients and 1994 non-AAD participants constituting 12 studies that used the cutoff value of 500 ng/ml, the sensitivity was 0.952 (95% CI 0.901–0.978) and the specificity was 0.604 (95% CI 0.485–0.712) (Table 2, Fig. 3B).

Sensitivity analysis based on the three high-quality reports comprising 1481 subjects suggested that sensitivity was 0.971 (95% CI 0.919–0.990) and specificity was 0.532 (95% CI 0.297–0.753) (Table 2, Fig. 3C, e-Fig. 2). These sensitivity and specificity figures in the sensitivity analysis did not greatly differ from those derived from the 12 studies above.

### Positive and negative likelihoods ratios

We estimated PLR and NLR from data of 12 studies that used a cutoff value of 500 ng/ml. This yielded PLR of 2.4 (95% CI 1.8–3.3) and NLR of 0.079 (95% CI 0.036–0.172). According to Grimes *et al.*, PLR in the range of 2–5 represents a small increase of probability when test is positive, and NLR <0.1 represents large decrease of probability when test is negative^43^.

Sensitivity analysis using data of three high-quality studies almost replicated PLR and NLR.

### Positive and negative predictive values

**Figure 4:**
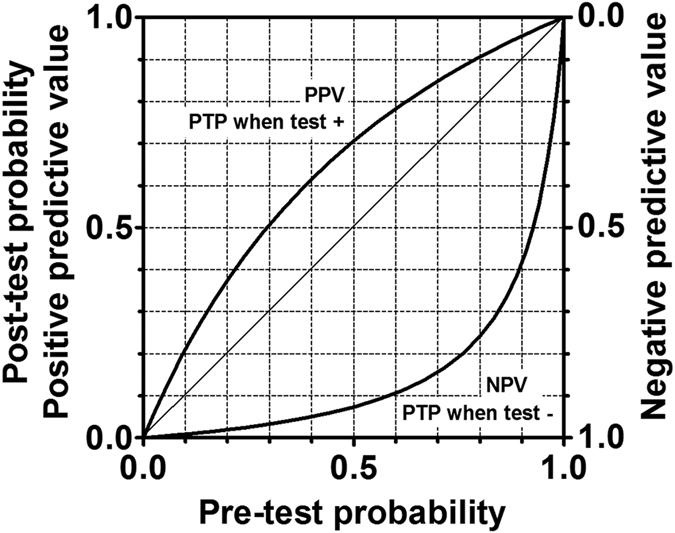
Post-test probability and predictive values. PPV: positive predictive value. NPV: negative predictive value. PTP: post-test probability. Diagonal line indicates completely meaningless test. PPV and NPV were estimated from a sensitivity of 0.952 and a specificity of 0.604.

We estimated PPV and NPV across pre-test probability ranging from 0% to 100% (Fig. 4).

### Classification of AAD

**Figure 5:**
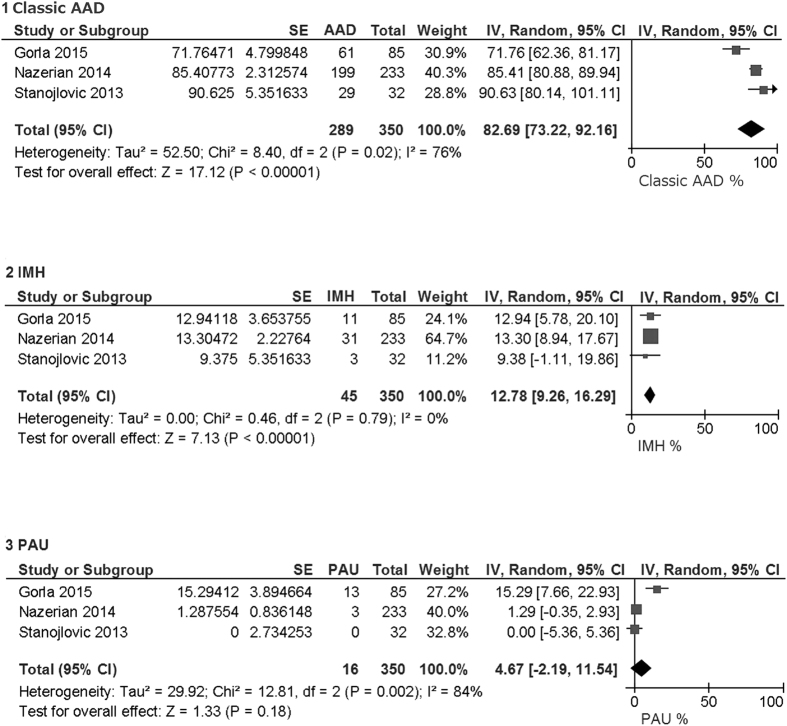
Classification of acute aortic dissection (AAD). Number of patients were counted regardless of D-dimer level. Total is sum of classic AAD, intramural hematoma (IMH), and penetrating aortic ulcer (PAU). Pooled percentage was estimated using inverse variance method and random-model.

Three studies presented numbers of patients with classic AAD, intraluminal hematoma, and penetrating aortic ulcer^16^,^20^,^29^. Classic AAD, intraluminal hematoma, and penetrating aortic ulcer constituted 83% (95% CI 73–92%), 13% (95% CI 9–16%), and 5% (95% CI 0–12%), respectively, of broad meaning of AAD (Fig. 5).

## Discussion

To our knowledge, the current study is the first systematic review and meta-analysis evaluating the diagnostic test accuracy of D-dimer for AAD using solid methodology and a sufficient number of original studies and subjects (Table 3). This was achieved by a careful study search and the use of a hierarchical model, and sensitivity analyses. Even though many meta-analyses concerning the same topic have been reported, they have had some common flaws: using univariate analysis, evaluation of studies with high risk of bias and with high applicability concern not thoughtfully, using a range of cutoff values collectively to estimate the pooled sensitivity and the pooled specificity, and limited numbers of original studies and subjects. In the current analysis, we confirmed that D-dimer has good sensitivity and moderate specificity using solid methodology when the cutoff value is 500 ng/ml (Table 2). D-dimer appears suitable for AAD and acute aortic syndrome rule-out without diagnostic imaging in patients with sufficiently low pre-test probability (Fig. 4).

A case-control study by Weber *et al.* in 2003 was a milestone in this area^30^. Until 2003, blood tests had played a very minor role in diagnosis of AAD. Weber *et al.* measured D-dimer levels of 10 AAD suspected cases, 14 definite AAD cases, and 35 patients who had chest pain that was caused by other causes than AAD. They used a cutoff value of 500 ng/ml because the cutoff value to exclude venous thrombosis in their hospital was 500 ng/ml. With this cutoff value, the sensitivity was 100% and specificity was 68.6% for AAD. Since then, numerous observational studies had re-confirmed the high sensitivity and the moderate specificity of D-dimer for AAD with the same cutoff value. Elevation of the D-dimer level reflects the fibrinolytic activity in responses to thrombosis of the false lumen and activation of the extrinsic pathway of the coagulation cascade with an injured aorta^12^,^30^. Among AAD cases, a highly elevated level of D-dimer also reflects the anatomical extent of the dissection^30^.

Nazerian *et al.* reported a retrospective cohort study evaluating the largest number of subjects with suspected AAD to assess the diagnostic accuracy of D-dimer for AAD^20^. They classified suspected AAD cases into three categories based on AAD risk score, score = 0, = 1, and ≥2. As pretest probability that was accessed by the risk score increased, PPV increased and NPV decreased. However, sensitivity and specificity were not greatly differed in all risk score groups. Across all score groups, the sensitivity was 0.983 and the specificity was 0.359. They reported higher sensitivity and lower specificity than our summary estimates of the sensitivity of 0.952 and the specificity of 0.604 (Table 2). This is a typical trade-off between sensitivity and specificity. Nazarian *et al.* commented that older age and high rate of comorbidities in the observed population contributed to the high D-dimer level, the high sensitivity, and the low specificity^20^. Recently, an age-adjusted D-dimer cutoff value, which is defined as age ×10 in patients 50 years or older, has been used to rule out pulmonary embolism and deep venous thrombosis, which increases specificity without modifying sensitivity^44^,^45^. Application of the age-adjusted D-dimer cutoff for ruling out AAD probably increases the specificity, however, this requires further research.

Classic AAD, intramural hematoma, and penetrating atherosclerotic ulcer constitute the acute aortic syndrome. It is very difficult to distinguish the three diseases from each other, because they have mutually overlapping pathology and symptoms^3^. Aortic intramural hematoma refers to an aortic wall hematoma not accompanied by an intimal flap. This accounts for approximately 10% of acute aortic syndrome cases (Fig. 5)^3^,^4^. Aortic intramural hematoma is caused by the rupture of the vasa vasorum at the aortic wall or microtears in the intima, while the intimal rupture precedes the intramural cleavage for AAD. Intramural hematoma formation frequently occurs in the descending thoracic aorta. It can perforate through the intima and lead to AAD^3^,^4^. Atherosclerotic aortic ulcers are also commonly observed in the descending aorta. When progressed deep into the aortic wall, the ulcer can result in intramural hematoma, penetrating ulcer, and aneurysm formation. Older age, hypertension, dyslipidemia, and severe aortic atherosclerosis are common risk factors for AAD, intramural hematoma, and penetrating atherosclerotic ulcer^3^,^4^. Given the pathology, it is reasonable for the D-dimer level to be elevated also for intramural hematoma and penetrating atherosclerotic ulcer. Actually, Garla and Stanojlovic reported that D-dimer levels were usually elevated for intramural hematoma and penetrating atherosclerotic ulcer^16^,^29^, which slightly lowered the specificity of D-dimer for AAD. Pulmonary embolism, deep venous thrombosis, systemic inflammation, and post-operation situation are also well known cause of D-dimer elevation, which leads to false positive in non-AAD population.

Even though the sensitivity and NPV of D-dimer are very good, D-dimer may overlook AAD^46^. False negative D-dimer are often observed for a young population, short dissection length, and thrombosed false lumen^11^,^17^.

A limitation of the current analysis is that most of the included studies had a high risk of bias or high applicability concerns. While some non-high-quality studies, namely studies by Sakamoto *et al.* and Shao *et al.* presented extraordinary low sensitivity (Fig. 2), three high-quality studies reported results compatible with previous reviews (e-Fig. 2)^9^,^10^. Anyway, the results of sensitivity analyses focusing on the high-quality report were consistent with those from all studies. Next, we evaluated numerous D-dimer assay methods collectively. Nonetheless, we observed only very weak heterogeneity during DOR evaluation (Table 2). The age dependency of DD is another limitation. We hope future researches will deal with the diagnostic test accuracy using age-adjusted DD cutoff values to predict AAD.

In conclusion, we performed this systematic review and meta-analysis of 22 studies with 5000 subjects to evaluate of the diagnostic test accuracy of D-dimer for AAD using a hierarchical model. DOR of 30.7 and AUC of 0.950 indicated that D-dimer has very good overall accuracy. Sensitivity of 0.952 and NLR of 0.079 meant that D-dimer <500 ng/ml largely decreases the possibility of AAD. Specificity of 0.604 and PPV of 2.4 meant that D-dimer >500 ng/ml moderately increases the possibility of AAD. Sensitivity analyses confirmed the robustness of our results.

## Additional Information

**How to cite this article**: Watanabe, H. *et al.* Diagnostic test accuracy of D-dimer for acute aortic syndrome: systematic review and meta-analysis of 22 studies with 5000 subjects. *Sci. Rep.* **6**, 26893; doi: 10.1038/srep26893 (2016).

## Supplementary Material

Supplementary Information

## Figures and Tables

**Table 1:** Characteristics of included studies.

Author (Year)	Country	Study design	Recruit setting	Reference test	Index test	Cutoff ng/ml	High quality
Akutsu^11^	Japan	pCohort	s/o AAD, r/o AMI. CCU	e-CT	Roche, Cardiac d-dimer sysetem	500	No
Eggebrecht^12^	Netherlands	Case-control	Control: PE, AMI, non-cardiac chest pain	TEE, Angio, CT, MRI	Dade Behring, D-Dimer Plus	500	No
Ersel^13^	Turkey	rCohort	s/o AAD. ED	e-CT	Dade Behring, quantitative immunoturbidimetric assay	246	No
Fan^14^	China	pCohort	s/o AAD. Admitted	TTE, TEE, CT, MRI	Tina-quant	500	No
Giachino^15^	Italy	pCohort	s/o AAD. ED	e-CT	STA LIATEST D-DI	500	Yes
Gorla^16^	Germany	rCohort	Chest pain. Admitted	TTE, TEE, e-CT, MRI, Angio	Innovance D-dimer	500	No
Hazui^17^	Japan	Case-control	Control: AMI. Admitted to critical care center	e-CT	Roche, Latex agglutination	900	No
Levcik^18^	Czech	rCohort	Acute chest pain. Admitted	CT, TEE, Angio, Autopsy	INNOVANCE D-Dimer assay, Liatest D-DI, Coamatic D-Dimer, D-Dimer plus	500	No
Li^19^	China	rCohort, CA	Not specified	e-CT	Not specified	500	Yes
Nazerian^20^	Italy	rCohort	s/o AAD. ED	CT-angio	Hemosil D-dimer HS, STA LIATEST D-DI	500	Yes
Ohlmann^21^	France	Case-control	Control: s/o AAD but later r/o AAD, Admitted	TEE, CT, MRI	Sta-Liatest D-DI immunoturbidimetric assay	400	No
Okazaki^22^	Japan	Case-control	Admitted for cardioembilic stroke	e-CT, plain-CT	LIAS AUTO d-dimer	8700	No
Peng^23^	China	pCohort	Chest pain. ED	CT-Angio	ELISA	2110	No
Reeps^24^	Germany	Case-control	Control: Chronic progressive type B dissection	CT-Angio, PET/CT	Not specified	500	No
Sakamoto^25^	Japan	Case-control	Control: PE/AMI	e-CT	LIAS AUTO d-dimer neo	5000	No
Sbarouni^26^	Greece	Case-control	Control: chronic aortic aneurysm, normal subject, Admitted	TTE, TEE, CT	Vidas, D-dimer ELISA	700	No
Shao^27^	China	pCohort	Chest/back/abdominal pain. Admitted	TTE, TEE, CT, MRI	Tina-quant D-dimer	500	No
Spinner^28^	Germany#	Cohort	Acute chest pain r/o STEMI. ICU	TEE, CT, Angio	Roche, Latex agglutination	300	No
Stanojlovic^29^	Serbia	Cohort, CA	Not specified	TTE, TEE, CT	Automated chemical analysis	500	No
Weber^30^	Austria	Case-control	Control: ICU case with chest pain r/o AAD	TTE, TEE, CT, MRI, Angio	Tina-quant assay	500	No
Xue^31^	China	pCohort	Chest pain, s/o AAD.	TEE, CT, MRI	Sta-Liatest D-DI immunoturbidimetric assay	400	No
Yoshimuta^32^	Japan	Cohort	TIA or ischemic stroke w/o chest symptom. ED	e-CT	Sekisui, Latex agglutination	6900	No

<Country> #: written in German language.

<Design> pCohort: prospective cohort. rCohort: retrospective cohort. CC: case-control. CA conference abstract.

<Recruit setting> AAD: acute aortic dissection. AMI: acute myocardial infarction. PE: pulmonary embolism. AAS: Acute aortic syndrome. STEMI: ST elevated myocardial infarction. TIA: transient ischemic attack. ED: emergency department. ICU: intensive care unit. s/o: suspected of. r/o: ruled out. AAD subjects for cases of case-control studies were default and not described.

<Reference test> CT: computed tomography. e-CT: enhanced CT. TTE: trans-thoracic echocardiography. TEE: trans-esophageal echocardiography. Angio: angiography. MRI: magnetic resonance imaging. PET: positron emission tomography.

<High quality> A study that had neither a high risk of bias nor a high concern regarding applicability and that used a cutoff value of 500 ng/ml was regarded as a high quality report.

**Table 2:** Summary of diagnostic accuracy by D-dimer for acute aortic dissection.

	All studies regardless of the cutoff value	Studies with the cutoff value of 500 ng/ml	High-quality reports
Studies	22	12	3
Acute aortic dissection	1140	833	402
Controls	3860	1994	1079
Diagnostic odds ratio	*28.5* (*17.6*–*46.3*) *I*^*2*^ = *17.4%*	30.7 (17.0–55.2) I^2^ = 7.7%	30.4 (17.2–53.7) I^2^ = 0%
AUC	*0.946* (*0.903*–*0.994*)	0.950 (0.847–1.000)	0.954 (0.909–1.000)
Sensitivity	Not available	*0.952* (*0.901*–*0.978*)	0.971 (0.919–0.990)
Specificity	Not available	*0.604* (*0.485*–*0.712*)	0.532 (0.297–0.753)
Positive likelihood ratio	Not available	*2.4* (*1.8*–*3.3*)	2.1 (1.4–3.9)
Negative likelihood ratio	Not available	*0.079* (*0.036*–*0.172*)	0.055 (0.018–0.177)

Brackets indicate 95% confidence interval.

High-quality reports: A study that had neither a high risk of bias nor a high concern regarding applicability and that used a cutoff value of 500 ng/ml was regarded as a high-quality report.

AUC: area under hierarchical summary receiver operating characteristics curve.

Main outcomes concerning diagnostic accuracy are written in *italics*. The others are results from sensitivity analysis.

**Table 3:** Summary of systematic reviews and meta-analyses evaluating the diagnostic accuracy of D-dimer for acute aortic dissection.

Author (Year)	Studies	Subjects	Model	Cutoff (ng/ml)	Quality assessment	Sensitivity analysis	Diagnostic odds ratio	AUC	Sensitivity	Specificity
Sodeck^10^	16	437	Random-effect	100–900#	QUADAS	Done	21.27	0.94	0.97	0.59
Marill^7^	11	541	Fixed-effect	500	NA	NA	NA	NA	0.94	0.95
Brown^50^	7	744	Not specified	500	NA	NA	NA	NA	0.97	0.56
Shimony^6^	7	744	Random-effect	500	QUADAS	NA	NA	NA	0.97	0.56
Shao^29^	9	1337	Random-effect	500	NA	NA	NA	0.88	0.89	0.68
Cui^8^	5	743	Random-effect	170–5000#	NOQAS	Done	NA	0.92	0.945	0.691
Asha^9^	4	1557	Random-effect	400–500#	QUADAS/STARD	NA	NA	NA	0.980	0.419
Watanabe	22	5000	Hierarchical	246–8700#	QUADAS-2	Done	28.5	0.946	NA	NA
Watanabe	12	2827	Hierarchical	500	QUADAS-2	Done	30.7	0.950	0.952	0.604

#using a range of cutoff values collectively.

QUADAS: the Quality Assessment of Diagnostic Accuracy Studies.

QUADAS-2: the Revised Tool for the Quality Assessment of Diagnostic Accuracy Studies.

STARD: the Standards for Reporting of Diagnostic Accuracy.

NOQAS: the Newcastle-Ottawa Quality Assessment Scale.

NA: not assessed.

AUC: area under (hierarchical) summary receiver operating characteristics curve.
