# Supplementary material for: Diagnostic test accuracy of D-dimer for acute aortic syndrome: systematic review and meta-analysis of 22 studies with 5000 subjects
Source: Sci Rep. 2016 May 27;6:26893. doi: 10.1038/srep26893 (PMC4882530; doi:10.1038/srep26893)
Supplement: Supplementary Information [file srep26893-s1.doc]

**Diagnostic test accuracy of D-dimer for acute aortic syndrome:**

**systematic review and meta-analysis of 22 studies with 5000 subjects**

**<<Supplementary File>>**

**Authors**

^1)^Hiroki Watanabe, ^1)^Nobuyuki Horita, ^1)^Yuji Shibata, ^2)^Shintaro Minegishi, ^3)^Erika Ota, ^1)^Takeshi Kaneko.

^1)^Department of Pulmonology, Yokohama City University Graduate School of Medicine, Yokohama, Japan.

^2)^Department of Cardiology, Yokohama City University Graduate School of Medicine, Yokohama, Japan.

^3)^Department of Health Policy, National Center for Child Health and Development, Tokyo, Japan.

**e-Appendix 1. Electronic search formulas.**

We used following formulas for electronic search.

Pubmed without limitation: ("D dimer"d OR "D-dimer" OR "fibrin fragment D") AND (dissection OR "acute aortic syndrome") AND (sensitivity OR specificity OR "predictive value" OR "likelihood" OR "true positive" OR "true negative" OR "false positive" OR "false negative" OR diagnostic OR diagnosis).

EMBASE: ('D dimer'/exp OR 'D dimer' OR 'D-dimer'/exp OR 'D-dimer' OR 'fibrin fragment D'/exp OR 'fibrin fragment D') AND ('dissection'/exp OR dissection OR 'acute aortic syndrome'/exp OR 'acute aortic syndrome') AND (sensitivity OR specificity OR 'predictive value'/exp OR 'predictive value' OR 'likelihood' OR 'true positive' OR 'true negative' OR 'false positive' OR 'false negative' OR diagnostic OR 'diagnosis'/exp OR diagnosis).

Cochrane Library, title/abstract/keyword search without limitation: ("D dimer" OR "D-dimer" OR "fibrin fragment D") AND (dissection OR "acute aortic syndrome").

Web of Science Core Collection advanced search without limitation: TS=("D dimer" OR "D-dimer" OR " fibrin fragment D") AND TS=(dissection OR "acute aortic syndrome") AND TS=(sensitivity OR specificity OR "predictive value" OR "likelihood" OR "true positive" OR "true negative" OR "false positive" OR "false negative" OR diagnostic OR diagnosis).


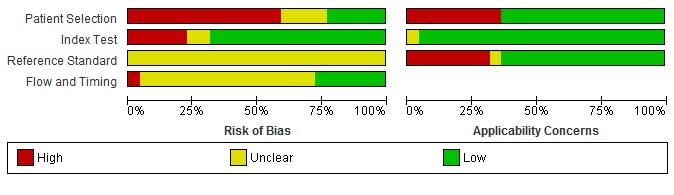
**e-Figure 1. Risk of bias summary.**

e-Figure 2. A paired forest plot by D-dimer for acute aortic dissection in high-quality
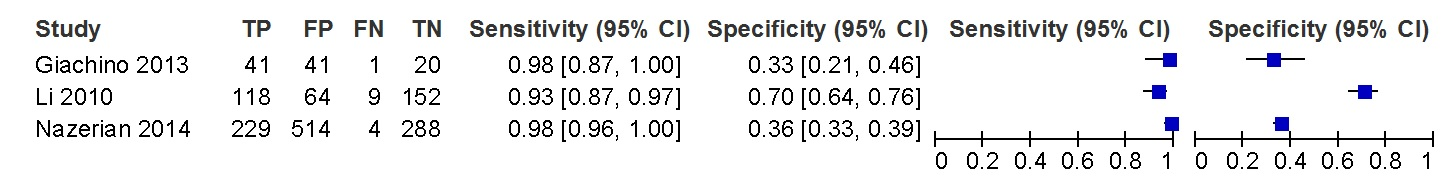
studies.
